# In the Face of a Pandemic: “I Felt the Same as When the War Started”—A Qualitative Study on COVID-19 Vaccine Acceptance in Bosnia and Herzegovina

**DOI:** 10.3389/ijph.2023.1606411

**Published:** 2023-10-13

**Authors:** Nina Bosankic-Cmajcanin, Sanjin Musa, Selvira Draganovic

**Affiliations:** ^1^ Psychology Program, International University of Sarajevo, Sarajevo, Bosnia and Herzegovina; ^2^ Public Health Institute of Federation of Bosnia and Herzegovina, Sarajevo, Bosnia and Herzegovina

**Keywords:** COVID-19, barriers, drivers, vaccine uptake, vaccines

## Abstract

**Objectives:** To conduct qualitative study with different target groups in Bosnia and Herzegovina in order to explore their views on barriers and drivers for COVID-19 vaccination, and to see if and how barriers and drivers vary between urban and rural locations, and different professional roles.

**Methods:** The theoretical framework underpinning the study is the capability-opportunity-motivation (COM-B) behavior change framework, which has been adapted to monitor vaccine related behavior and attitudes. Data was collected from June to September 2022 through moderated discussions in focus groups. The total of 162 participants participated in 16 focus groups.

**Results:** Among the key barriers to successful immunization identified across target groups were insufficient knowledge about vaccines, pandemic fatigue, concerns about the rapid development of the vaccine and its effectiveness, lack of confidence in the healthcare system. Some of the main drivers of vaccination against COVID-19 were confidence in science and expert recommendations.

**Conclusion:** The COVID-19 immunization policy undergoes continuous changes, as do the pandemic prospects; we encourage further research to track the evolution of vaccine related attitudes, inform immunization policy, and create evidence-based interventions.

## Introduction

Safe and effective vaccination is a key public health intervention to control the COVID-19 pandemic. It is estimated that COVID-19 vaccinations prevented 14.4 million (95% Crl 13.7–15.9) deaths from COVID-19 across 185 countries and territories in the first year that vaccines were available [1]. Nevertheless, COVID‐19 vaccines have posed a unique set of challenges to vaccine acceptance.

COVID-19 vaccine hesitancy has been found to differ significantly across countries and among key groups, posing a threat to vaccination campaigns [2]. Negative perceptions of safety, trust in the science behind vaccine development, and vaccine efficacy were the most consistent correlates of COVID-19 vaccine hesitancy, as well as government mistrust, while other factors varied by country and included personal experience with COVID-19 and demographic characteristics [3].

Healthcare workers (HCWs) were a priority group for COVID-19 vaccination worldwide [4, 5]. Moreover, HCWs are considered a reliable source of information and can significantly influence patients’ decisions about vaccination. Therefore, if attitudes regarding COVID-19 vaccination are negative among HCWs, it is likely that such attitudes would be negative among the general public as well [6]. Determinants of vaccination hesitancy among HCWs are mainly related to concerns about the vaccine’s side effects or lack of scientific information about the vaccine [7]. Specific to western Balkan countries, literature indicates that, in general, HCWs moderately believed that COVID-19 vaccines were a beneficial and safe intervention, would successfully reduce disease burden, and that socio-demographic characteristics might not be particularly relevant factors of vaccination intention [8].

According to data from the World Health Organization (WHO), almost 2 million doses of COVID-19 vaccines were administered in Bosnia and Herzegovina (BiH) (population 3.2 million) by the end of January 2022, of which almost 850,000 people were fully vaccinated [9]. It is important to note that the official data likely underestimate COVID-19 vaccination coverage, due to the vaccination of BiH residents in neighboring countries as a result of significant COVID-19 vaccine supply constraints during the first half of 2021, as well as challenges calculating accurate denominators. Authorities in the state procured COVID-19 vaccines that were developed using a variety of different platforms, initially mostly vector-based and inactivated vaccines.

To develop effective recommendations for policies, it is important to understand the drivers and barriers to vaccination [10], especially in key professions such as HCWs and teachers; as well as the lowest-coverage groups, such as people aged 18–24 and the working-age population (e.g., individuals aged 25–65). The first objective of the current study was to explore the views of target groups regarding barriers and drivers for COVID-19 vaccination, as well as on recommending vaccination to patients (for HCWs), organized around four main themes: knowledge and health literacy concerning COVID-19 vaccines and vaccination; attitudes and intentions towards COVID-19 vaccines and vaccination; social support and social norms for COVID-19 vaccines (and recommending vaccination - for HCWs), and; access to COVID-19 vaccination.

The second objective of the study was to analyze the data to see if and how barriers and drivers varied between urban and rural locations, and different professional roles (teachers, medical doctors, nurses).

## Methods

### Study Design

The study was conducted from June 2022 to September 2022 on the territory of Bosnia and Herzegovina, in both entities, in four phases [1]: protocol preparation [2], field work organization [3], data collection, and [4] data analysis. Data was collected through moderated focus group discussions, with focus groups comprised of participants from the same target group. Focus groups were selected as the data collection method because they allow participants to freely exchange opinions, that is, the exchange of opinions can be stimulated by the presence of others. The group discussions lasted between 60 and 90 min on average, and were moderated by a trained researcher. The study was conducted in the local (Bosnian/Croatian/Serbian) language. Interviews were recorded with participants’ permission. The focus groups discussions followed a guide, which contained semi-structured questions, and anonymity was guaranteed to facilitate participant comfort, and willingness to speak openly, that is, to freely share their opinions and give open and honest answers. The protocol and discussion guide were developed by researchers with expertise in qualitative methodology (NBC and SM), and they are based on the WHO Rapid qualitative research to increase COVID-19 vaccination uptake—A research and intervention tool [11]. The theoretical framework underpinning the study and analysis is the Capability-Opportunity-Motivation (COM-B) behaviour change framework, which has been adapted and used to monitor vaccine-related behaviour and attitudes and has also been used for designing a discussion guide [10, 11]. The COM-B framework identifies the interrelated factors of individual (physical and psychological) capability, physical and social opportunities, and motivation as influences on individuals’ vaccination related behaviors, providing a holistic approach to exploring barriers and opportunities associated with individual health behaviors.

### Data Collection and Analysis

Focus group participants were identified using convenience and snowball sampling. A total of 162 participants participated in 16 focus groups at 10 locations, intentionally selected by researchers to represent various occupations, ages, administrative and geographic locations (both urban and rural), to provide a diversity of views and experiences. Detailed demographic information about the study participants is provided in Table 1 below. Of note, turnout among women was considerably higher than it was among men.

**TABLE 1:** Demographic characteristics of participants (N = 162) (Bosnia and Herzegovina, 2023).

	Doctors N (%)	Nurses N (%)	Teachers N (%)	Adults 18–24 N (%)	Adults 25–65 N (%)	Total N (%)
Sex						
Male	1 (5.6)	2 (7.7)	3 (11.1)	11 (30.6)	27 (49.1)	44 (27.2)
Female	17 (94.4)	24 (92.3)	24 (88.9)	25 (69.4)	28 (50.9)	118 (72.8)
Age						
18–24	0	1 (3.8)	0	36 (100)	6 (10.9)	43 (26.5)
25–50	15 (83.3)	22 (84.6)	20 (74.1)		35 (63.6)	92 (56.8)
51–65	3 (16.7)	3 (11.5)	7 (25.9)		14 (25.5)	27 (16.7)
Type of settlement						
Urban	9 (50.0)	18 (69.2)	19 (70.4)	17 (47.2)	34 (61.8)	97 (59.9)
Rural	9 (50.0)	8 (30.8)	8 (29.6)	19 (52.8)	21 (38.2)	65 (40.1)
Vaccination status						
First dose	2 (11.1)	3 (11.5)	1 (3.7)	1 (2.8)	3 (5.5)	10 (6.2)
Second dose	15 (83.3)	18 (69.2)	13 (48.1)	13 (36.1)	33 (60.0)	92 (56.8)
Not vaccinated	1 (5.6)	5 (19.2)	13 (48.1)	22 (61.1)	19 (34.5)	60 (37.0)
Confirmed COVID-19						
Yes	10 (55.6)	17 (65.4)	14 (51.9)	11 (30.6)	20 (36.4)	72 (44.4)
No	8 (44.4)	9 (34.6)	13 (48.1)	25 (69.4)	35 (63.6)	90 (55.6)
Entity						
Federation of Bosnia and Herzegovina	18 (100)	17 (65.4)	17 (63.0)	25 (69.4)	46 (83.6)	123 (75.9)
Republic of Srpska	0	9 (34.6)	10 (37.0)	11 (30.6)	9 (16.4)	39 (24.1)

Participation in the study was voluntary. Ethical approval for conducting the study was provided by the Ethical Committee of the International University of Sarajevo. Potential benefits are that participants had the opportunity to share views, concerns and experiences that will influence the considerations of a strategy formulated as a result of this study. The participants received a gift certificate worth EUR 10 for their participation in the study.

The focus groups discussions were recorded by the moderator/researcher (NBC). A verbatim transcript of all audio recordings was made in the local language. All files and recordings were stored in password protected computers. Only the study team members had access to the recordings and transcripts. Copies of the recordings were deleted after all anonymous transcriptions were completed.

No identifiable data were collected. The collected demographic data on participants included age, sex, geographic location (rural/urban, entity), profession, information on confirmed COVID-19 infection, and vaccination status.

Researchers (SM, NBC, SD) analysed the transcripts using a deductive coding framework [12], which comprised a modified COM-B behavioural framework. Data were coded and tabulated in a rapid assessment matrix in three deductive phases. During the final phase, after creating the barrier tables, the researchers identified different sub-themes from the tables for each modified COM-B factor/four main themes. Tables with barriers were subsequently consolidated for the general population—aged 18–24 and 25–65, while HCWs and teachers are presented in separate tables.

## Results

Target groups’ attitudes on COVID-19 vaccination barriers and drivers are presented below. Barriers are further presented in tables by the following COM-B factors/four main themes: [1] knowledge and health literacy concerning COVID-19 vaccines and vaccination; [2] attitudes and intentions towards COVID-19 vaccines and vaccination; [3] social support and social norms for COVID-19 vaccines (and recommending vaccination—for HCWs); [4] access to COVID-19 vaccination. Due to significantly lower number of drivers compared to barriers, they will be presented in the text only and will not be included in the tables. Of note, differences according to location (urban/rural) and professional group (doctors/nurses) are highlighted.

### Barriers

#### Healthcare Workers

All HCWs mentioned how difficult and stressful the period during the pandemic was, an experience they compare to the one they had during the war in Bosnia and Herzegovina (1992–1995). Almost all of them emphasized the lack of knowledge about vaccines at the pandemic onset. There was a general impression among HCWs that nurses are less informed about vaccines than doctors, which is also a potential barrier for promotion of vaccination among patients. Most HCWs participated in the teams that carried out vaccination to patients, but the majority were not familiar with the Vaccination Plan or results of the immunization programme.

A few HCWs expressed being hesitant when it comes to COVID-19 vaccination, and emphasized a lack of social support in the professional environment. Most mentioned professional burnout, as well as pandemic fatigue, both in general and in the media.

Most participants mentioned vaccination hesitancy among patients, emphasizing older individuals were the most interested in receiving the vaccine.

Most HCWs did not express any infection concerns for themselves or their patients. A number of HCWs, mainly nurses, mentioned fear about vaccination safety. A subgroup of nurses who worked in COVID hospital wards, however, did not report any hesitancy.

An overview of barriers to positive vaccination behaviour and illustrative quotes organized by COM-B factors are provided in Table 2.

**TABLE 2:** Barriers and illustrative quotes by COM-B—Health workers (Bosnia and Herzegovina, 2023).

Barriers to positive vaccination behaviour (themes and sub-themes from revised matrix)	Quote and ID of the focus group
Th1:Knowledge and health literacy	I administered the first vaccine, took it over, and I administered it for a month, two, three, I do not even know, it was a disaster, “I do not know”, no matter who I ask, “I do not know”, so I called the institute, then information service, asking what am I gonna do and how; Pfizer was the best for me, you need to dissolve a certain amount, then times ten… stress, I got sick for 15 days, all that stress made me sick. (G2.1 -MS1, nurse)
Sth1.1: Lack of knowledge about vaccines at the pandemic onset. Although they now state that they do not need any new information, updated contents should be provided. Nurses have lower knowledge level than doctors	As they say, you always hear something new; after two and a half years, we learned a lot, whoever wanted to, could learn a lot here, and as they say, any new information you hear, it comes in handy later, it’s never out of place, but I think, we who… that we had heard a lot (G2.3-MS2, nurse)
Sth1.2: Insufficient knowledge about the COVID-19 vaccination programme and its results	We do not know anything in particular about the plan, the programme, we followed everything that was current. (G2.3-MS2, nurse)
Th2:Access to vaccination	
Sth2.1: Lack of awareness of the need for operational teams at HI	
Th3:Social support for vaccination	…Even among us, health workers, there is hesitation, how many times have I told my colleagues who were in meetings, please, just get dressed and go see what it looks like in the COVID wards; let’s not talk about you doing nothing; not everyone was directly involved in that, we tried to change people. (G2.3-MS1, nurse)
Sth 3.1: Some health workers hesitate and refuse vaccination in the professional environment	It was produced in a short period of time, there was not enough time to see the side effects, I mean, we did not even have time to think, it was like a war for me, that’s how I am, that’s how it is, you can’t let people die because you are afraid of stroke caused by AstraZeneca. (G1.2-DR1, doctor)
Sth 3.2: Pandemic fatigue in general and in the media	I worked with COVID patients; you are standing there and working; I am looking at a Covid-positive man and I have nothing but a surgical mask, and I examine him, and listen to him, and he leaves, and now I have the little free time, and I sit down and open the x portal and I get heart palpitations from nervousness, from fear of corona. I’m afraid of it as I read about it, and for example, when I examine someone who is Covid-positive, I’m not afraid of them at all. I say shut down TV… (G1.2-DR5, doctor)
Th4:Attitudes and intentions	The current strains are too mild, that’s one thing, and the other thing is, we have a new one every year for the flu and we adapt it to the strains, and this vaccine is really not adapted now, I see no reason for further vaccination. (G1.2-DR2, doctor)
Sth4.1:They do not perceive any importance to the continuation of vaccination and re-vaccination of themselves and their patients	The vaccine helped with all that, I believe it did, and then came these two strains that really have nothing to do with it. I advise all patients against vaccination now, to wait for something new, if there is anything. (G1.2-DR3, doctor)
Sth4.2: Professional burnout	For the first 2 years, working with so much adrenaline, the desire to help, to solve it, I feel it only now, I feel sick, but only for the last few months, I can’t stand the sound of mobile phones, only now do I see how much we were traumatized by it.(G1.2-DR2, doctor)
Sth4.3:Fear about vaccination safety	There was fear… I believed that the Chinese vaccine was the best, because they have the virus and they produced it, I thought they would make a genuine vaccine; those are old-style vaccines, I thought, I′ do not care what and how, I knew I would not want Pfizer or Moderna, because they are new vaccines and it’s an experiment again. (G1.2-DR4, doctor)
Sth4.4:Nurses who have not worked in COVID wards are more hesitant when it comes to vaccination	When I was COVID-positive, it was not so bad for me, I think that the body is fine and there is no need for it, because I do not see any purpose; even the colleagues who got vaccinated also got Covid. (G2.2 -MS1, nurse)
Sth4.5:Preference for natural immunity boost and acquiring immunity through infection	My view is that the virus is as it is, but that the hosts are crucial for the outcome of the clinical picture, overall health condition at the time of the infection, that is, contact with that virus. (G2.1 -MS3, nurse)

#### Teachers

Regarding teachers who participated in the focus groups, most pointed out fear and uncertainty as dominant emotions during the pandemic, with some noting that the measures implemented to prevent the spread of infection were excessive, as well as the need for normalization of life.

Most participants were familiar with the types of vaccines available, but did not sufficiently display knowledge about vaccines and the country’s COVID-19 vaccination programme. Many teachers from rural areas had an extremely negative attitude towards vaccines. These teachers described media as being their main source of information. Some participants from rural areas overemphasized the boost of natural immunity through a healthy diet, while others emphasized the acquisition of immunity through infection and exposure. Some participants also mentioned wanting to hear a “different opinion” about vaccines in the public space. Some noted perceiving that vaccines were not available in time, and described getting vaccinated in a neighbouring country. Some waited for the vaccines to arrive in BiH, and described being vaccinated without any problems at a local healthcare facility.

Teachers stated that vaccination depends on one’s individual decision, and some mentioned problems related to lack of confidence in the healthcare system, as well as hesitancy of some HCWs to recommend vaccination. Insufficient support for vaccination from family, friends, and some HCWs also emerged as themes.

Most teachers described no longer being worried about infection, and did not consider vaccination to be necessary anymore. Barriers to vaccination included concerns about the rapid development of the vaccines, questionable effectiveness of the vaccines, and fear of adverse reactions.

Barriers to positive vaccination behaviour and illustrative quotes are provided in Table 3.

**TABLE 3:** Barriers and illustrative quotes by COM-B—Teachers (Bosnia and Herzegovina, 2023).

Barriers to positive vaccination behaviour (themes and sub-themes from revised matrix)	Quotes and ID of the focus group
Th.1.: Knowledge and health literacy	So, no, until it is safe; and it was very funny when someone said - but you do take a pill; I do, but it has probably been in use for 50, 100 years, I guess it has passed some tests, I know that it has some side effects, but this was made overnight, people, overnight. (G3.2 - PR1, teacher)
Sth1.1.: Insufficient knowledge about vaccines and the COVID-19 vaccination programme	We have such an army in our body, our organism, the human body is so perfectly designed, I would say, truly a masterpiece of nature and now, after all those studies, you suddenly tell me that my immunity is not good, if you are healthy in a healthy body, would not it be healthy to think that, although corona is a new thing, let’s not be afraid, I trust that my body knows how to deal with it. (G3.2-PR3, teacher)
Sth1.2.: Preference for natural immunity boost and acquiring immunity through infection (rural area)	I have never seen in the media that the two sides were confronted so that we can hear both, and then decide; but only one side was presented and, come on, I have a feeling that if it had been more transparent, maybe a lot more people would have had confidence. (G3.3-PR2, teacher)
Sth1.3.:Highlighting the importance of portraying polarized opinions about vaccination in the media	
Th2: Access to vaccination	We waited for 3 months, vaccines did not arrive, and so we went to Serbia, my sister, brother-in-law and I. My mother got vaccinated here, when it was her turn, when they called her from the infirmary. (G3.3-PR5, teacher)
Th2.1:Delay in starting the vaccination for a part of the population	
Th3: Social support for vaccination	People were divided, in families even, some say do this, some say do that, you absolutely must be vaccinated or do not dare to get vaccinated, one had to decide for themself… (G3.2.- PR3, teacher)
Sth3.1.:Insufficient support for vaccination in the environment, from family, friends and health workers	…you go there to get the vaccine, they just ask you if you are sick, and that’s it, they administer the vaccine, and nothing else. Not even ‘what symptoms do you experience’, nothing. No explanations whatsoever. I do not think it’s been examined enough, even the doctors themselves can’t explain it because they do not know enough either. (G3.1-PR4, teacher)
Sth3.4.:Lack of confidence in the healthcare system	
Th4:Attitudes and intentions	And there is this sadness because even if you decide to get vaccinated out of good intention, and I respect that, in the end you will probably experience a great disappointment when you see that you did not help yourself or others as much because it does not last that long and in the end it does nothing special for you and you are not safe. (G3.2 - PR2, teacher)
Sth4.1.:They no longer consider COVID-19 vaccination necessary and/or adequate	The virus is weakening, there are fewer people on ventilators, less hospitalizations, this indicates that we can tolerate it better. No need for vaccination. (G3.3-PR3, teacher)

#### General Population (Youth, Adults)

Younger individuals who participated in focus groups reported stress due to online classes and lack of contact with teachers and peers during the pandemic.

Regarding COVID-19 vaccination, there was a lack of knowledge of detailed information about vaccines and vaccination against COVID-19 among younger focus group participants, including about: vaccine development, different types of vaccines available, protection offered by the vaccines, safety, and re-vaccination. The majority of younger participants pointed out that vaccination is no longer a topic of interest in their social circles, and mentioned the excessive coverage of the pandemic in the media.

A few participants noted that the beginning of vaccination was delayed, and that they were vaccinated in a neighbouring country. Those who were vaccinated in the country did not mention any problems with access to vaccination.

Many younger participants reported that they were not vaccinated, particularly those from rural areas. The main barriers to vaccination described were: lack of trust in COVID-19 vaccines, concerns about the speed of vaccine development and safety, and low priority for vaccination against COVID-19 because they do not consider the disease to be serious. Some younger participants noted that they preferred to acquire immunity by contracting the infection. Some younger participants cited a lack of trust in the healthcare system, as well as conspiracy theory beliefs.

Participants aged 25–65 reported negative experiences during the pandemic, including feelings of fear and uncertainty. They compared the situation to the war in Bosnia and Herzegovina. Those who lived in rural areas, especially in the countryside, coped with the anti-pandemic measures more easily. Lack of support for vaccination in the social environment stood out even among adults, as did fatigue related to the pandemic and its presence in the media.

Concerns about the rapid development of vaccines, as well as their safety and effectiveness, emerged as barriers to vaccination uptake, which was more significant in rural areas. Almost all participants currently had a low perception of infection risk, indicating low re-vaccination probability. Several participants mentioned lack of confidence in the healthcare system and a tendency to believe in conspiracy theories.

Barriers to positive vaccination behaviour and illustrative quotes are provided in Table 4.

**TABLE 4:** Barriers and illustrative quotes by COM-B—General population (Bosnia and Herzegovina, 2023).

Barriers to positive vaccination behaviour (themes and sub-themes from revised matrix)	Quotes and ID of the focus group
Th.1.: Knowledge and health literacy	I do not know if this vaccine can be called a vaccine, because as far as I know you get the vaccine once and that’s it, and here there is pressure to get it once, then again, and then there’s little risk, but get the third one just in case… (G4.1 – M1, youth)
Sth1.1.: Insufficient knowledge about vaccines and the COVID-19 vaccination programme	I did not get vaccinated because of the lack of information, because, I do not know, I’m young, and I do not know what the consequences of the vaccine are, and I did not want to. (G5.3 –O2, adults)
Sth1.2.: Preference for natural immunity boost and acquiring immunity through infection (rural area)	Is there a plan here? I have not seen any plan in our system, it is unknown to me. (G5.2 –O4, adults)
Sth1.3.:Highlighting the importance of portraying polarized opinions about vaccination in the media	That’s why we should invest more in prevention than in some vaccines out there that I have my doubts about. And when you are healthy, you do not need anything, you can fight it and win. (G5.1 – O1, adults)
	The global immunity of the entire population on planet earth will be acquired (through exposure), … though, COVID will probably never end, because you can still find a case of swine flu that was popular 10 years ago, it still exists, you can’t just stop it forever. I just think that the number of cases will decrease over time and that people will simply stop talking about it, just as they stopped talking about Ebola, and bird flu, and swine flu, and everything. (G4.2- M5, youth)
	I’s not fair, some smart people, they were not heard, they were just removed from TV…(G5.3 – O2, adults)
Th2: Access to vaccination	We had the opportunity to register online, and I was one of the first to register, and they called me to come a year after I had been vaccinated in Serbia. (G5.2-O3, adults)
Th2.1:Delay in starting the vaccination for a part of the population	
Th3: Social support for vaccination	Everyone in my family got vaccinated, mom, dad, sisters got Pfizer, and they say they regretted taking it, because somehow, they are constantly sick, they feel tired, they come down with flu more often than before; I’m glad that I have not been vaccinated. (G5.3 – 03, adults) I have a cousin who worked in the Covid hospital up in X and I had a chance to talk to him 7 days ago and he says: I was there, I saw all the symptoms; he was isolated from his family, he isolated himself because he is a health worker, so as not to pass it all on to them, and he says that these events (poor health condition) (…) are consequences of the vaccine… (G5.2 –O6, adults)
Sth3.1.:Insufficient support for vaccination in the environment, from family, friends and health workers	I am not informed because I was not interested in that, for one simple reason. I really was not interested in it, but I really, because I think that none of us, not even medics, not even doctors can know about it enough due to the fact that, even though they have finished university and everything, Covid is a new thing, so it is unknown to them and to us. (G4.1- M2, youth)
Sth.3.2.:Vaccination is not a topic of interest among youth	As for attitudes concerning vaccines, there are never-ending debates, vaxers and anti-vaxers, autism - not autism, you get corona - you do not get corona, the types of vaccines, the traditional ones, the modern ones, the ones… I do not know what they are called, it is so annoying… I think that education from verified source was the only important thing; if (someone is interested in the topic), if they are going to Google it, they should not Google it from a forum site, but from the World Health Organization’s website or another valid source and that’s it, I think. (G4.4- M2, youth)
Sth3.3.:Pandemic fatigue in general and excessive coverage in the media	Media spread fear and phobia intensively and deliberately. So, even if you’re watching a movie and really want to relax, you’ll see the ‘be responsible’ slogan somewhere in the corner. So, those were some games and I should say manipulation, to the extent that all your daily thoughts go down to Covid. (G5.4 – O6, adults)
Sth3.4.:Lack of confidence in the healthcare system	I do not trust our healthcare system at all. First, because the messages coming from that segment are very often contradictory; you receive one message now, and in 2 weeks you will get a different message. (G5.2-O1, adults)
Th4:Attitudes and intentions	Once, twice, how many times? What for? I’m done. (G4.2 – M4, adult)
Sth4.1.:They no longer consider COVID-19 vaccination necessary and/or adequate	I think we have bigger problems that we are not solving, especially when it comes to medicine, because to us, when someone on TV says any number of Covid deaths, it sounds a lot to us, whether it’s 100 people or one million people, it’s just it sounds like a lot, because it is said with such an intonation, that it just sounds terrible to us; however, look at how many people die from cancer, how many die from blood pressure, how many die in traffic, so many people die every day, so many young people die, and nobody does anything about it (G4.2 – M1, adult)
Sth4.2.: Low priority for vaccination against COVID-19, because they do not consider the disease to be serious. Low perception of infection risk	You read about the side effects on various portals, and it scares you a little, for example, you will not be able to have children, they want to reduce the number of people, there are too many of us on the planet, all kinds of information. I did not read much about it at first, actually media created a hype over it, that we should not get vaccinated. Again, some pushed for it, there were various stories, there was some fear of the vaccine, and that’s why I did not take it. It was developed too quickly, I do not know. (G5.3 –O2, youth)
Sth4.3:Concerns about rapid development of vaccines, they question the effectiveness of vaccines and fear adverse reactions	I would say that maybe we are too used to playing with our minds, whether it’s corona or over the years, for me this is all 1984, George Orwell and stuff, in terms of how far and how it all goes, how much someone actually plays with us; today it is corona, tomorrow it will be something new (G4.3- M3, youth)
Sth5:Conspiracy theory beliefs	The media, they were paid to talk only about that, they talk too much, the figures are too high, and all the information is false, I do not believe it; so, they are paid by the globalists to do that. (G5.4 – O2, adults)

### Drivers

#### Healthcare Workers

Facilitators for COVID-19 vaccination receipt were aligned with participant confidence in science and professional recommendations of competent institutions, particularly among doctors.

There were some lectures organized through the zoom application. I remember one lecture, professor x explained each vaccine, its composition… We all basically read it, we all read about it ourselves online and then commented among ourselves… what type, what can you use, contraindications, our epidemiologist helped us a lot, she was like an ace up one’s sleeve, we called her in case of any ambiguity… (G1.2-DR3, doctor)

If scientists say that it is good, I am not competent to say that it is not. There are always side effects, even to paracetamol, of course there will be some to the vaccine… (G2.1-MS1, nurse)

In addition, the majority of participants noted that vaccination is necessary for pandemic control and prevention of severe disease outcomes, and that they considered this obligation as their professional duty.

Vaccines have always saved the world, smallpox was eradicated, right? Then let's get this going, people. (G1.2-DR3, doctor)

My mom said to me - take a sick leave, I can't take sick leave! It’s just like in the war - it’s my obligation. And you have to give your best, so it was very turbulent and difficult, you work under a uniform, with gloves, plaster sticks to you, you can't place IV properly, people die. (G2.2-NS3, nurse).

HCWs emphasized the importance of exchanging experience and information within the professional community, through both formal and informal communication channels:

Our group helped me a lot, a Viber group was created, I don't know who was the initiator, was it this colleague who is originally from BiH and works at the Mayo Clinic, or were there several people, and Zagreb and Belgrade and Sarajevo were all included, and then, that group really included everyone, from ‘small doctors’ who had just graduated, to the wow professors teaching different subjects, and you could really read about many different things there (…) we also had our own group, there were several, we had a health center’s group, which helped us a lot, for testing, and for vaccination, for information, and for the clinical picture, and transportation. I mean, what was done there, that group, family medicine, including ambulance, every information, we sent X-ray pictures, clinical pictures, received information... (G1.2-DR5, doctor)

Good organization of vaccination with operational teams within healthcare institutions emerged as one driver of vaccination uptake, but potential failure of those teams in the future (and not only when it comes to vaccination of HCWs against COVID-19, but also other diseases, e.g., flu) may be an aggravating factor for access to vaccination.

It was well organized, especially for health workers, and we all acted according to the plan, we were registered, names... We had priority, we got it right away, although it was AstraZeneca... and all kinds of (negative) things were said about it at the time…...(G2.1-DR1, doctor)

#### Teachers

Vaccination as a necessity for health protection and pandemic control, trust in science, and vaccination as a social norm emerged as vaccination drivers among teachers:

Simply, I have two options, either to take it regardless of the fact that it was quickly produced and that it has not yet been tested enough because it is the only way to do something for myself, to try to protect myself, or not to take it, to side with the group that says it's all a conspiracy theory and wait for my destiny (...), we have to trust scientists (...) so I decided to get vaccinated. (G3.3-PR1, teacher)

A number of participants mentioned the organization of vaccination for teachers:

In the beginning, when we made a list, and people whose names were on the list were called, as there were no vaccines at the beginning (for general population), and later, you could go and get it whenever you wanted... (G3.2-PR1, teacher)

In addition, participants who received the vaccination mentioned the following reasons for vaccination uptake: working conditions, travel, vulnerable family members, and their health.

#### General Population (Youth, Adults)

Among vaccinated participants aged 18–24, mostly among those who lived in urban areas, the motives for vaccination were their own health, travel possibilities, and workplace requirements:

At first, I didn't want to get vaccinated, and then my parents decided to get vaccinated, and when I saw that it was done without any problems, and some restrictions on accessing certain places and countries were imposed, I realized that my health condition could only get worse, I could contract the virus, so I decided to get vaccinated, these are both reasons (G4.1 – M3, youth)

Participants aged 25–65 who had received the vaccination noted the necessity of vaccination to protect their health and control the pandemic as motivating factors:

So, all those who were vaccinated will probably in some way strengthen their immune system in case of infection, they will have somewhat milder symptoms and will spread the infection less, so you have that one positive effect of this planetary vaccination and I think it worked, you know. Imagine if humanity had not been vaccinated at all, I think that the scale of the pandemic would have been much wider and much bigger; I think that on a global scale, I don't know the number of vaccinated people, 8-9 billion, maybe some 30-40% of people have already been vaccinated, this slowed down the pandemic spread and probably caused the virus to ‘weaken’. (G5.4 – O5, adults)

For some, vaccination was considered a social norm:

More or less, our view of all this is a civic one, that means we have to follow the instructions, we have to follow the management system, the rule of law and so on, and we have to behave like citizens. Whether it's right or wrong, whether it's true or not, that's more or less irrelevant now, that's just my opinion. (G5.4 – O3, adults)

In addition, participants described access to vaccination at their local healthcare centre as being mostly appropriate:

So, the process itself was simple, you apply, they call you, ask if I want re-vaccination, when the vaccine supplies arrive; Pfizer for example, in my case it was Pfizer, they call you and let you know that Pfizer supplies arrived. (G5.1 –O1, adults)

Those who had been vaccinated noted the importance of the presence of an informed HCW who elicits high levels of trust.

During the COVID period, I went to my family doctor who has been treating me for 15 years; I trust him, and I asked him if I should, he said yes; I asked which one? He says it doesn’t matter. This was our conversation about the vaccine. (G5.2-02, adults)

## Discussion

To the best of our knowledge, this study was among the first to examine barriers and facilitators to COVID-19 vaccination uptake among individuals in Bosnia and Herzegovina. Taken together, the results from this study suggest barriers to COVID-19 vaccination in this population, including: insufficient knowledge about the vaccines, the COVID-19 vaccination programme and its results, delays in starting the vaccination program, insufficient support for vaccination in private and professional settings, pandemic fatigue exacerbated by media coverage, concerns about the rapid development of the vaccine, its effectiveness and fear of side effects, low perception of infection risk, lack of confidence in the healthcare system, overemphasis on boosting natural immunity through healthy diet, preference for acquiring immunity through exposure to virus, the influence of polarized thoughts about vaccination in the media, and belief in conspiracy theories.

Further, our results identified the following as being drivers of COVID-19 vaccination: confidence in science and expert recommendations from competent institutions, the exchange of experience and information within the professional community through both formal and informal communication channels, a sense of obligation, and consideration of vaccination as a professional duty, particularly among doctors. HCWs and other target groups also emphasized the necessity of vaccination for pandemic control, health protection, prevention of severe disease outcomes, and its role as a social norm.

Our research was conducted with a population that has first or secondhand experience with the war in Bosnia. The pandemic experience, especially for HCWs, often triggered memories of this war, leading to feelings of fear, helplessness, and a lack of trust in public information. These factors may have hindered coping with the pandemic and resulted in the identification of more barriers than drivers among participants.

Both the barriers and drivers identified in our study align with the results of similar studies in Bosnia and Herzegovina [13–15]. These studies indicated that a small number of citizens, mainly those with higher education, higher income levels, and the elderly population, opted to get vaccinated against COVID-19, often motivated by the goal of achieving collective immunity. On the other hand, the majority of citizens expressed hesitancy or outright refusal to vaccinate [15]. Factors noted in the literature as contributing to and promoting hesitancy to vaccinate against COVID-19 include limited knowledge about vaccines and relatively pessimistic attitudes about vaccines [16]. Findings from other countries [17–20] also highlight factors associated with vaccine refusal and hesitancy, including concerns about vaccine safety, negative narratives related to various vaccines and their effectiveness, and individual’s personal knowledge about vaccination.

Furthermore, literature suggests that daily exposure to various misinformation about COVID-19 and vaccines led to confusion, anxiety, and vaccine mistrustful [21–23]. Most participants in the current study, regardless of their respective groups, mentioned the negative influence of media during the pandemic. Both the general public and HCWs were exposed to misinformation about COVID-19 and the development of related vaccines via media [24]. This created additional confusion and polarized thinking among people, which contributed to various conspiracy theories, which were also a barrier to vaccination. Research indicates that belief in conspiracy theories, especially those related the origin of the pandemic, is a significant barrier to vaccination [25–28]. Results of our study corroborate the presence of such beliefs.

Although perceived vaccination risk appears to have had a greater influence on individual’s willingness to vaccinate than the perceived risk of COVID-19 [29], studies also indicate that those who perceive a higher COVID-19 risk are less hesitant about vaccines. Many such individuals received or intend to receive the COVID-19 vaccine [29–33], as do those who express confidence in healthcare institutions and HCWs [33]. People more willing to get vaccinated tend to have higher education levels, express strong confidence in the vaccine, maintain a positive attitude toward the vaccine, fear COVID-19, personally (i.e., perceive COVID-19 risk), and have had contact with suspected or confirmed COVID-19 patients [33]. Similar to other European studies [34–37], our participants - doctors and HCWs who worked in COVID-19 wards - also expressed greater willingness to get vaccinated.

In our study, a significant portion of teachers, similar to HCWs, no longer (as of summer 2022) expressed significant concerns about COVID-19 infection and did not perceive vaccination as necessary, except for vulnerable groups. However, during the early stages of the pandemic, they did experience anxiety and raised concerns about the rapid development and effectiveness of vaccines. Similar studies involving teachers have yielded comparable results [38–40].

The ability to travel was one of the main COVID-19 vaccination drivers in our study across different groups. This aligns with findings from other studies where freedom of movement, travel opportunities, encouragement from family and friends, and positive past vaccination experiences have been identified as significant drivers for vaccination [41, 42].

The COVID-19 pandemic and immunization policies have undergone continuous changes, leading to shifts in public attitudes towards COVID-19 vaccines. This study contributes to the knowledge of key barriers and drivers for vaccination in Bosnia and Herzegovina during the summer of 2022. As vaccination efforts persist in Bosnia and Herzegovina, addressing key barriers for HCWs remains critical. Accordingly, interventions should involve regular updates of information on official websites, the organization of symposiums and immunization training programs, as well as fostering cooperation and engagement with medical chambers and associations. Recommendations and encouragement for vaccination (e.g., through official letters) by competent ministries, public health institutes, associations, professional bodies, and certification of education on immunization by professional chambers should also be taken into consideration. One of the ways to exchange information and knowledge is through online learning communities, using mobile phone applications (Viber and WhatsApp).

This study has some limitations. The study was conducted within a short time frame during the summer vacation season, which posed challenges in achieving the desired participation level due to project constraints. This was, however, compensated for by including more locations with a lesser number of people per focus group than initially envisioned. More women participated in our focus groups than did men. However, in the case of HCWs and teachers, this discrepancy can be partly attributed to the higher representation of women in these professions. Regarding reflexivity, we made efforts to remain aware of our potential biases throughout the research process. While we continually questioned and edited materials to mitigate bias, our prior and recent experiences, assumptions, and beliefs about COVID-19 vaccines may have still influenced the research process.

Good organization of access to vaccination, sufficient time for vaccination, wide availability, and flexible working hours at vaccination points during any pandemic should continue to be standard practices. Additionally, vaccination reminders and invitations via phone calls, texts or emails are crucial. Vaccination promotion campaigns seem to also have a positive effect [43], and over the past 2 years, with the support of international agencies, health authorities in Bosnia and Herzegovina conducted comprehensive campaigns targeting various demographics. The importance of communication about immunization and presence of a HCW with effective communication skills are evident from our study. With the main focus now predominantly on individuals at risk of contracting disease, different communication channels should be used, as well as work with individuals and communities through dialogue, with mutual respect and appreciation of different opinions. Given that the WHO declared an end to COVID-19 as a public health emergency 9 months after the data collection, we encourage further research to monitor the evolution of vaccine related attitudes, inform immunization policy, and develop evidence-based interventions.
